# Identify Risk Pattern of E-Bike Riders in China Based on Machine Learning Framework

**DOI:** 10.3390/e21111084

**Published:** 2019-11-06

**Authors:** Chen Wang, Siyuan Kou, Yanchao Song

**Affiliations:** Intelligent Transportation System Research Center, Southeast University, Nanjing 211189, China; 220183009@seu.edu.cn (S.K.); 220173180@seu.edu.cn (Y.S.)

**Keywords:** e-bike rider, crash risk, machine learning, traffic violation

## Abstract

In this paper, the risk pattern of e-bike riders in China was examined, based on tree-structured machine learning techniques. Three-year crash/violation data were acquired from the Kunshan traffic police department, China. Firstly, high-risk (HR) electric bicycle (e-bike) riders were defined as those with at-fault crash involvement, while others (i.e., non-at-fault or without crash involvement) were considered as non-high-risk (NHR) riders, based on quasi-induced exposure theory. Then, for e-bike riders, their demographics and previous violation-related features were developed based on the crash/violation records. After that, a systematic machine learning (ML) framework was proposed so as to capture the complex risk patterns of those e-bike riders. An ensemble sampling method was selected to deal with the imbalanced datasets. Four tree-structured machine learning methods were compared, and a gradient boost decision tree (GBDT) appeared to be the best. The feature importance and partial dependence were further examined. Interesting findings include the following: (1) tree-structured ML models are able to capture complex risk patterns and interpret them properly; (2) spatial-temporal violation features were found as important indicators of high-risk e-bike riders; and (3) violation behavior features appeared to be more effective than violation punishment-related features, in terms of identifying high-risk e-bike riders. In general, the proposed ML framework is able to identify the complex crash risk pattern of e-bike riders. This paper provides useful insights for policy-makers and traffic practitioners regarding e-bike safety improvement in China.

## 1. Introduction

As a convenient, economical, energy-saving, and environmentally-friendly travel tool, electric bicycles (e-bikes) can not only meet the needs of people traveling short or medium distances, but they are also be able to propel the development of the green sustainability concept. At present, China has become the largest e-bike producer and consumer country around the world. According to statistics, e-bike ownership in China has sharply increased from 58 thousand to 29.96 million, from 1998 to 2014 [1,2]. Experts also predict that the ownership of e-bikes may be in a continuous growth condition with the technological innovation and policy improvement of e-bikes in the future [3].

However, increased e-bike ownership also brings a lot of challenges for traffic safety [4]. Yao claimed that the number of crashes reached nearly 56.2 thousand, with an approximately 8.6% annual increase until the year of 2017 in China [5]. Feng et al. revealed the fact that although the total crash rate in China was in a declining state, the crash rate of e-bikes was still a rising trend [6]. Many studies have also shown that the driving behavior of e-bike riders is non-identical to the regular bike riders, that they tend to be more aggressive and careless. Moreover, compared with other drivers, e-bike riders are more prone to violate traffic rules [7]. Those findings suggest that identifying the risk pattern of e-bike riders is important and necessary. Previous literature has revealed many findings regarding the contributory factors of e-bike crash risks, including e-bike violation behaviors, e-bike demographics, and the psychological factors of e-bike riders. However, there is limited literature, to our best knowledge, for identifying the complex risk pattern of e-bike riders.

Thus, in our study, the risk pattern of e-bike riders in Kunshan, China, was deeply examined. At-fault e-bike riders were considered as a high-risk group, while other e-bike riders were used as a non-high-risk group. A number of violation-related features were developed, and four tree-structured machine learning models were utilized to capture the complex risk patterns of e-bike riders. This study is expected to provide policy-makers and traffic practitioners with useful insights into e-bike safety.

## 2. Literature Review

Previous studies have concluded that the demographics, aberrant riding behaviors, and environmental conditions can be selected as predictors of the driving risk of e-bike riders [8]. For example, Haustein et al. found that = age has a negative correlation with e-bike riding safety. They also found that male riders were more likely to encounter riding risk [9]. A study implemented in China indicated that male and aggressive riders were more likely to increase riding risk [10]. Petzoldt et al. claimed that riding e-bikes at a high speed can induce a higher crash risk [11]. Du proved that unregulated riding behavior and riding without a helmet are prone to increase riding risk [12]. Hertach et al. also found that poor roadway conditions would increase riding risk [13]. In addition, some literature has also found that the crash risk of e-bike riders was related to whether they were registered or not [14].

Regarding the crash modeling technique, most previous literature adopted traditional statistical methods to identify crash-related contributing factors. For example, Cherry et al. used simple mathematical statistical indicators to analyze the riding behavior of e-bike riders [15]. Although this method can analyze the driving behavior of e-bike riders, it requires a large amount of historical data and failed to predict the driver’s future driving behavior quantitatively [16]. In addition, some mathematical analysis models have also been introduced into the study of the riding behavior of e-bike riders. For example, the logit model was selected to analyze the risk behavior of e-bike riders [17]. Guo et al. adopted a probit model to analyze the factors related to e-bike riders’ riding risk [18]. Wang et al. applied the regression model to identify the association between the riding risk and the related factors [19]. Although these studies reasonably identify some risk aspects of e-bike riders, a comprehensive risk pattern recognition of e-bike riders is still lacking. In particular, a complex risk pattern (e.g., non-linear relationships) was not found.

In general, considerable accomplishments have been achieved in e-bike-related safety research. However, few studies have been found to focus on exploring the complex risk pattern of e-bike riders, based on a machine learning framework.

## 3. Study Design

This study was conducted based on e-bike violation records and crash records during the years between 2015 and 2018, throughout the Kunshan City of Jiangsu Province in China, which were collected by the local traffic police department. There were a total of 242,030 e-bike riders identified in both of the two records. The combined dataset contains the demographic, violation-related information, and crash-related information for each e-bike rider, by matching their previous violation and crash records.

Previous studies have shown that the crash rate and fatality rate of e-bike riders were significantly higher than for other vehicle drivers [20]. Once a crash occurs, it is very likely to induce road congestion [21,22]. These findings indicate that when an e-bike accident occurs, it is a significant hazard to both the rider and the traffic condition around him/her. Therefore, it is essential to identify e-bike riders who are prone to crashes. According to previous literature, at fault e-bike riders in crashes were largely considered as a high-risk group [23], while those not at-fault were often used as quasi-induced exposure [24]. There were some arguments on the quasi-induced exposure method about whether not at-fault drivers are representatives of the total population. However, it has been agreed that at-fault drivers are a specific group with a higher crash risk.

Thus, in this study, e-bike riders were divided into two groups, namely: high-risk (HR) riders and non-high-risk (NHR) riders. Those with at-fault crash involvements were determined as HR, while all of the other riders (non-at-fault and no crash involvement) were considered NHR. After cleaning the origin data, 2605 e-bike riders were labeled as HR and 239,425 riders were labeled as NHR.

In order to identify the HR e-bike riders based on the machine learning methods, a number of features were developed. As for the demographics, the age groups were divided into four categories based on exclusive class intervals, namely, teenagers (<18 years old), young-aged riders (18~35 years old), middle-aged riders (35~65 years old), and old-aged riders (>65 years old), according to previous literature [25].

Temporal violation features were developed. For example, if one has two prior violation records during the morning peak-hour traffic in Kunshan (i.e., 07:00–09:00), the feature value for morning peak-hour violation will be coded as two. Spatial violation features were also considered. All of the violation locations were classified into the three groups (high, medium, and low) based on their prior cumulative violation frequency. For determining the high and low locations, 15 and 85 percentiles were used. For example, if one has three violation records at low-violation locations, the low-violation location feature value will be coded as three.

From the original violation dataset, the violation behavior features in the three years were directly extracted for each e-bike rider. For example, if one had two red-light running violation records, the corresponding feature value (i.e., type three: violating traffic signal) would be coded as two. Violation punishment-related features were also developed, including the frequency of violations, frequency of violation type, cumulative penalty, amount of violation penalty, and so on. Table 1 depicts a detailed explanation of the features extracted from the original dataset.

## 4. Methodology

**Figure 1:**
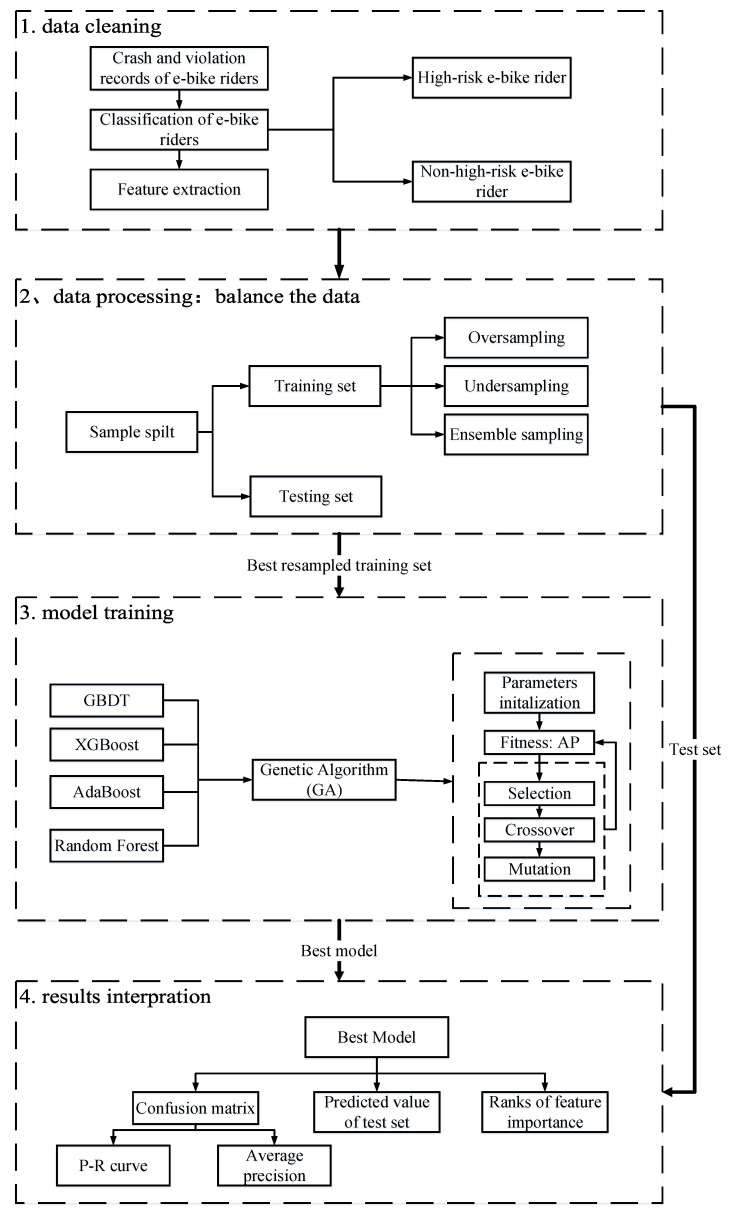
Technique flow chart. P–R—precision–recall; GBDT—gradient boosting decision tree; AP—average precision value.

In order to reasonably identify the risk patterns of e-bike riders, a systematic machine learning-based approach was proposed to deal with a number of data mining issues, including imbalanced datasets, machine learning model selection, hyper-parameter tuning, and model validation. These issues will be discussed in the following sub-sections in Section 4. Figure 1 briefly describes the technical flow of the approach.

### 4.1. Sampling Techniques

In the dataset, the number of NHR riders greatly exceeds the number of HR riders, which could cause over-fitting issues. Therefore, sampling techniques should be adopted in order to balance the training dataset. In this study, an over-sampling technique (i.e., synthetic minority oversampling technique (SMOTE)), an under-sampling technique (i.e., cluster-centroid method (CC)), and an ensemble method (i.e., balanced bagging classifier (BBC)) were compared. The average precision values (APs) were used as performance measures.

The under-sampling method adopted in this study is the cluster-centroid (CC) method, which makes use of a k-means algorithm to cut down the number of samples in a majority class. The SMOTE method was considered to be an easy and practical over-sampling method [26], the main idea of which is to synthesize new samples based on the K-nearest neighbors of the original samples in the minority class [27]. The ensemble method used in this study is the balanced bagging method, which combines both the sampling and classification techniques. This method first divides the imbalanced dataset into several subsets first. Then, the under-sampling technique and the classifying estimators will be applied to each subset. At last, the final result can be calculated by synthesizing the result of each subset.

**Figure 2:**
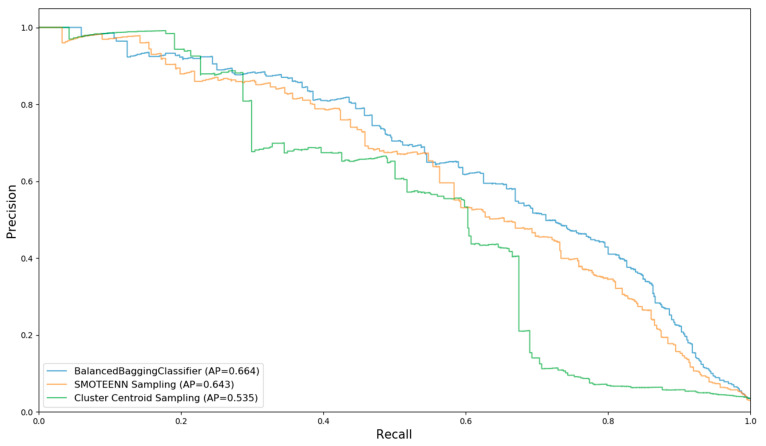
P–R curve of each sampling technique.

After balancing the training set with the above three sampling techniques, a random forest (RF) classifier was provided for the model training. Figure 2 shows the precision–recall (P–R) curves and AP values of the three sampling methods using RF.

From the figure, it can be concluded that the balanced bagging method has the best performance. Thus, it was chosen as the standard sampling method, based on which four different tree-structured machine learning models (RF, Adaboost, XGboost, and GBDT) were further trained and compared.

### 4.2. Model Training

In this study, four tree-structured machine learning methods were utilized for the model training, including the RF algorithm, AdaBoost algorithm with a decision tree, XGBoost algorithm, and the gradient boosting decision tree (GBDT). They are all ensemble machine learning algorithms based on decision trees, so that they are more interpretable than other machine learning models.

#### 4.2.1. Random Forest

The random forest model is an optimization of the decision tree-based algorithm. The random forest introduces two kinds of randomness, namely: (1) random selected samples and (2) random selected feature variables, which make the algorithm insensitive to noise and difficult to over fit. Therefore, the prediction precision of the random forest algorithm can be improved effectively.

The principle of this algorithm is to randomly extract *n* subsets from the dataset, and to randomly extract *m* features for each subset. Then, *n* incomplete decision trees can be established by the randomly selected samples and features. At last, the output result of each decision tree can be integrated, and then the final classification result of each sample is determined through means of voting.

#### 4.2.2. AdaBoost

The AdaBoost classifying technique was first discussed in 1995 by Schapire and Freund [28]. The main idea of AdaBoost is to combine the weak classifiers with suitable weights so as to form a strong classifier [29]. The weighted value can be calculated over multiple iterations. For example, if the sample is classified into the wrong class, its weights will be increased, and vice versa. If the weak classifier has a worse performance than the others, its weight will be reduced, and vice versa. In this study, the selected weak classifier in AdaBoost will also choose a decision tree. The steps of this algorithm can be concluded as follows:

1. Initialize weights $D_{1}=\left\{w_{11},\;w_{12},\;w_{13},\;\ldots ,\;w_{1n}\right\}$,
  $\mathrm{where},\;w_{1i}=\frac{1}{n},\;i=1,2,3\ldots n$
2. For m=1 to M:
  (1) Update training set ${\left\{\left(x_{i},\;y_{i}\right)\right\}}_{i=1}^{n}$ by weighted values;
  (2) Fit a decision tree $T_{m}$, namely:
    $$T_{m}\left(x\right):x\to \left\{-1,\;+1\right\}$$
  (3) Compute classification error $e_{m}$, as follows:
    $$e_{m}=\sum_{i=1}^{n}w_{i}I\left[T\left(x_{i}\right)\right]$$
    If $T\left(x_{i}\right)\neq y_{i}$, then, $I\left[T\left(x_{i}\right)\right]=1$; if $T\left(x_{i}\right)=y_{i}$, then $I\left[T\left(x_{i}\right)\right]=0$;
  (4) Compute weights $\beta_{m}$ of decision tree $T_{m}\left(x\right)$, as follows:
    $$\beta_{m}=\frac{1}{2}log\frac{1-e_{m}}{e_{m}}$$
  (5) Update the model, as follows:
    $$F_{m}\left(x\right)=sign\left(\sum_{m}\beta_{m}T_{m}\left(x;\;w_{m,i}\right)\right)$$
  (6) For i=1 to n, update weights $D_{m+1}$ of the training set, as follows:
    $$w_{m+1,i}=\frac{w_{m,i}}{z_{m}}exp\left\{-\beta_{m}y_{i}I\left[T\left(x_{i}\right)\right]\right\}$$
    Let $z_{m}=\sum_{i=1}^{N}s_{m,i}exp\left\{-\beta_{m}y_{i}I\left[T\left(x_{i}\right)\right]\right\}$.
3. Output $F_{M}\left(x\right)$.

#### 4.2.3. Gradient Boosting Decision Tree

As for GBDT, it generates a strong classifier by weighting the weak classifier. The purpose of this algorithm is to minimize the loss function. The loss function can be defined as $L\left(y,F\left(x\right)\right)=\log\left(1+e^{-yF\left(x\right)}\right)$, in which F(x) is the predicted value. The function of F(x) is $F\left(x\right)=\sum_{m=1}^{M}\beta_{m}h_{m}\left(x;\;s_{m,i}\right)$. To minimize the loss function, a residual term can be added to the prediction function *F*(*x*) in each iteration [30,31]. The steps of this algorithm can be concluded as follows:

1. Initialize model as constant *c*:
  $$F_{0}\left(x\right)=argmin\sum_{i=1}^{N}L\left(y,c\right);$$
2. For m=1 to M, as follows:
  (1) For i=i to N, compute the so-called pseudo-residuals, as follows:
    $$r_{im}=-{\left[\frac{\partial L\left(y_{i},\;F\left(x_{i}\right)\right)}{\partial F\left(x_{i}\right)}\right]}_{F\left(x\right)=F_{m-1}\left(x\right)};$$
  (2) Fit decision tree $h_{mi}$ to $r_{im}$ by training set ${\left\{\left(x_{i},\;y_{i}\right)\right\}}_{i=1}^{n}$;
    $$h_{mi}=\frac{y_{i}}{1+\exp\left(y_{i}F_{m-1}\left(x_{i}\right)\right)};$$
  (3) Calculate weights $\beta_{m}$ by minimizing the loss function L:
    $$\beta_{m}=argmin_{\beta }L\left(y,\;F_{m-1}\left(x\right)+\beta_{m}h_{mi}\right);$$
  (4) Update $F_{m}=F_{m-1}\left(x\right)+\beta_{m}h_{mi}$;
3. Output $F_{M}\left(x\right)$

#### 4.2.4. XGBoost

The last adopted algorithm in this study is XGBoost. The main idea of this algorithm is similar to GBDT. Both of them are trying to form a strong classifier by iterating the previous classifier. The differences between XGBoost and GBDT reflect in the following two aspects. First, the objective function of XGBoost is not just a loss function as defined in GBDT. It will add a new term Ω(h(x;s)) after the loss function, which is used to reduce the complexity of the decision tree. Secondly, in GBDT, the tree just fits the first order gradient of the loss function. But XGBoost will replace the first order gradient with both first order and second order gradients when generating a new decision tree in each iteration [32].

## 5. Model Results

### 5.1. Model Performance

**Figure 3:**
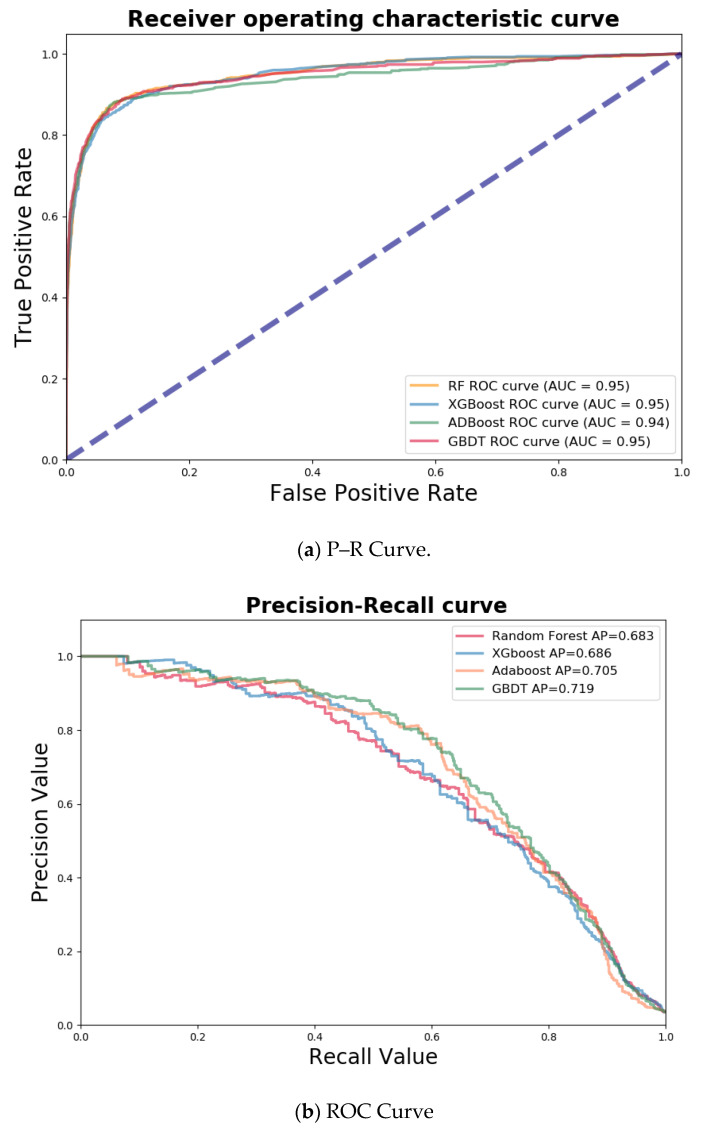
Estimation of the model performance. ROC—receiver operating characteristic.

In this study, 75% of the data were used for the model training, while the remaining 25% were used for the model testing. Figure 3 shows the receiver operating characteristic (ROC) curves and precision–recall (P–R) curves of the models. The ROC curve reports the false positive rate and true positive rate. The false positive rate was calculated as the ratio between the number of NHRs wrongly categorized as HR and the total number of actual NHRs. The true positive rate refers to the proportion of HR correctly categorized by the model. A P–R curve reports the precision and recall, which is often used to evaluate the model performance for an imbalanced dataset. The precision is the percentage of correctly predicted HR over all of the predicted HR. The recall is calculated in the same way as the true positive rate.

The four models all have a large area-under-curve (AUC), indicating that they are capable of identifying both the HR and NHR in general. According to the P–R curves, the average precision of the four machine learning models on the testing dataset were 0.683, 0.686, 0.705, and 0.719 (RF, XGBoost, AdaBoost, and GBDT, respectively). The results show that GBDT appears to be the best model, as it is able to better identify the HR of the e-bike riders.

Moreover, the thresholds can be further adjusted to obtain an even higher precision for the HR of e-bike riders, while lowering the recall. The testing dataset contains a total of 18,609 samples, in which the number of HR riders is 651, and the number of NHR riders is 17,958. The adjusted GBDT model prediction results are shown in Table 2, below.

### 5.2. Feature Importance

**Figure 4:**
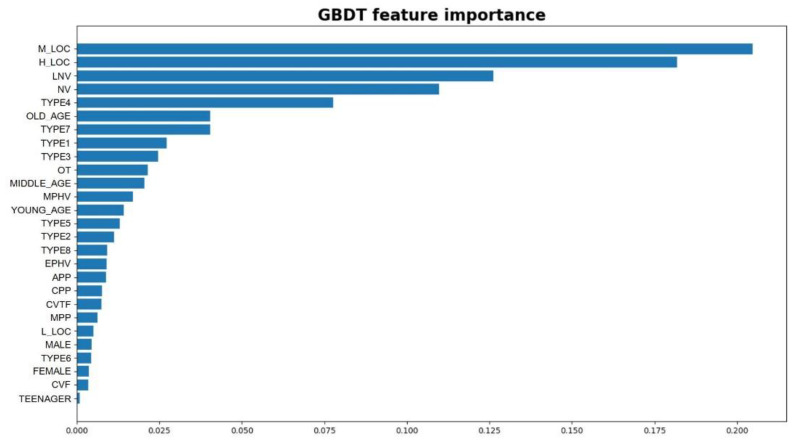
Feature importance extracted from the GBDT model.

As for the tree-structured machine learning models, the Gini index can be used to evaluate the feature importance. The higher the Gini index, the more importance a feature has. Figure 4, below, presents the importance ranking of all of the features, based on the GBDT model.

From the results of the feature importance, it can be seen that the top three most influential features are the medium-violation location (M_LOC), high-violation location (H_LOC), and late-night violation period (LNV). It is surprising to find that the spatial and temporal characteristics of the previous violation experience have the largest effects on the crash risk of e-bike riders. A possible reason could be that those features are related to risk exposure. Violation behaviors were also found to be important, such as aggressive riding (type 4), overloading (TYPE7), not riding on the non-vehicle lane (type 1), and red-light running (type 3). On the other hand, however, violation punishment-related features were not found to be as important as other violation features. As for demographics, older drivers were also found to be an important feature.

### 5.3. Partial Dependence

To deeply examine the complex risk patterns of e-bike riders, the partial dependence was calculated for each feature [31].

$$f_{x_{i}}\left(x_{i}\right)=E_{x_{-i}}\left[f\left(x_{i},x_{C}\right)\right]=\int f\left(x_{i},x_{C}\right)dP\left(x_{C}\right)\;(3)$$

where $x_{i}$ is the feature *i*, for which the partial dependence function needs to be calculated, and $x_{C}$ is the other features used in model *f*. The partial function is estimated by calculating the averages in the training data, based on the Monte Carol method, as follows:

$$f_{x_{i}}\left(x_{i}\right)=\frac{1}{n}\sum_{i=1}^{n}f\left(x_{i},x_{Ci}\right)\;(4),$$

where $x_{Ci}$ is the actual values of the feature set $x_{C}$, according to the dataset, and *n* is the number of instances in the dataset.

**Figure 5:**
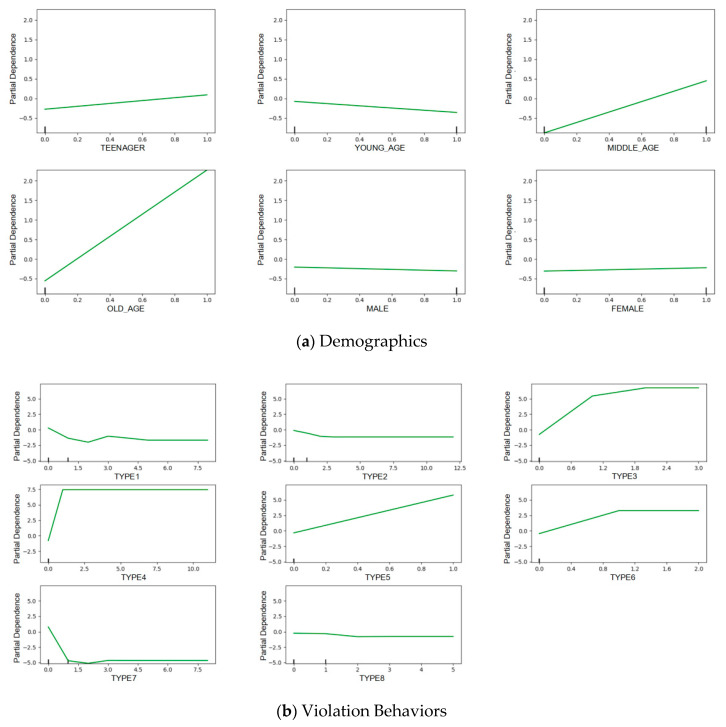

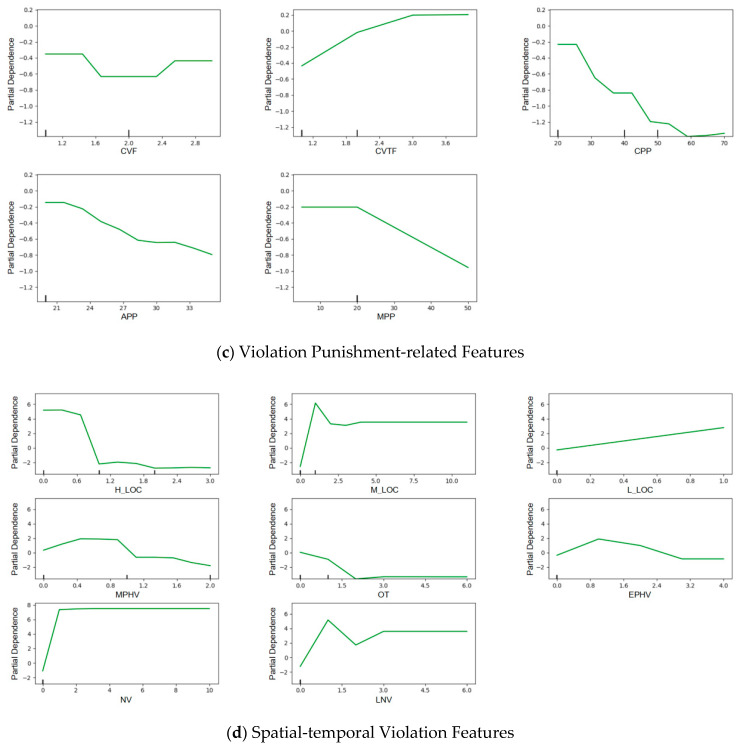
Partial dependence plots of the different features.

The major advantage of this method is to reveal complex relationships between each factor and outcome. As shown in Figure 5, for each graph, the *x*-axis indicates a feature, while the *y*-axis presents its partial dependence. It can be found that the effects of each feature tend to be non-constant across different levels. In doing so, a complex relationship between the features and outcomes can be properly identified.

It appears that gender has little effect on the risk pattern of e-bike riders (Figure 5a). There is a slight trend that females are more likely to be high-risk riders. Regarding age, it has been shown that older and middle-aged e-bike riders tend to have a higher crash risk. According to previous literature, middle-aged riders are more likely to be involved in a crash [18], because their cognitive ability regarding the surrounding roadway risk is weaker than young-aged riders. Older e-bike drivers were also found to be at high risk, as a result of their diminished physical capabilities [17].

From Figure 5b, the e-bike riders with more previous violation experiences of signal violation (type 3), aggressive riding (type 4), over-speed riding (type 5), and drunk driving (type 6), are more likely to be high-risk riders. Those with more experience in lane violation (Type 1) and overloading (type 7) tend to be in the low-risk group. These findings are interesting. Sometimes e-bike riders commit lane violations (i.e., not riding on the non-vehicle lane) may not be intentional, when the non-motorized lane is occupied by other vehicles or e-bikes. E-bike riders with more overloading violation could be more skilled riders. However, these violation behaviors still deserve further investigation.

From Figure 5c, e-bike riders with many previous violation penalty fees and points tend to be NHR riders. This indicates that riders with more penalty points and fees tend to be less aggressive and more cautious, based on the current violation penalty system. Thus, the current violation penalty system can be considered to effectively alter the dangerous driving habits of HR riders.

According to Figure 5d, e-bike riders with more previous violation experience at high-frequency violation locations tend to be NHR. These violate traffic rules at middle- and low-frequency violation locations tend to be HR. At low-frequency violation locations, most e-bike riders obey traffic rules because of their awareness of traffic enforcement, dangerous roadway conditions, and so on. Thus, those who still violated the rules could be either much more aggressive or have a lack of safety awareness, exposing themselves to dangerous situations. Moreover, the violation behaviors of e-bike riders at low-frequency violation locations could be unexpected for other road users, and more likely to cause crashes. E-bike riders with more violation experiences at night (i.e., night violation (20–24) and late night violation (0–6)) are more likely to be high-risk riders. During the night, visibility could be largely decreased for drivers, because of poor light conditions. Thus, e-bike riders are more likely to be involved in a crash with motor-vehicles when they violate traffic rules.

## 6. Conclusions

In this study, the complex risk pattern of e-bike riders was examined based on a systematic machine learning framework. Three-year crash/violation records were acquired from the Kunshan traffic police department. Based on the quasi-induced exposure theory, at-fault e-bike riders were considered as a high-risk (HR) group, while other e-bike riders (including non-at-fault and non-crash-involved) were determined as a non-high-risk (NHR) group. Demographics and violation-related features were created, and four tree-structured ML models were developed to differentiate the HR group from the NHR group. The following conclusions can be drawn:

(1) High-risk e-bike riders can be reasonably identified based on a machine learning approach. The major advantage of ML models is that they can better identify complex relationships between the features and e-bike riders’ riding risk (i.e., the complex risk pattern), which are not easily captured in traditional statistical methods. Explicable methods, such as tree-based ensemble methods, are highly recommended for such tasks.
(2) Spatial-temporal violation features were found to be important. Riders with more violation records in low- and medium-violation locations appeared to be high-risk riders. Meanwhile, those with more violation records during night were likely to be high-risk riders. Those features could be useful for identifying and characterizing high-risk e-bike riders.
(3) Riders’ violation behavior was found to be highly correlated with crash risk, as it is able to reflect riders’ risky riding habits and attitudes. Violation behavior features appear to be more effective than violation punishment-related features, in terms of capturing the risk pattern of e-bike riders.

In general, the proposed method appears to be a promising tool to identify the risk pattern of e-bike riders in China. Moreover, as such, potential high-risk e-bike riders can be early identified, and certain safety interventions can be applied on this group. For example, they can be mandatorily required to attend traffic school, or can receive safety warning messages routinely. In doing so, e-bike safety in China is expected to be improved in the future.

Admittedly, this research also has some limitations. First, it would be interesting to understand if/how the selected features could support the prediction of new e-bike riders that might have some features missing. Second, it would be more convincing to compare the proposed model to other statistical/machine learning models. However, to our best knowledge, this is the first paper to identify the complex risk pattern of e-bike riders based on machine learning models. Third, the full database containing all of the registered e-bike riders can be used to validate the model. The current database only contains those with either violation or crash records. However, according to quasi-induced exposure theory [24], the current model can still be considered as a reliable tool. We recommend that future research can focus on these directions.

## Figures and Tables

**Table 1:** The features extracted from the original dataset.

Type	Feature	Variables
**Demographic Information**	Gender	Male
		Female
	Age	Teenager (<18)
		Young age (18~35)
		Middle age (35~65)
		Old age (>65)
**Environmental Information**	Temporal violation features	Late night violation (LNV; 0–6)
		Morning peak hour violation (MPHV; 7–9)
		Evening peak hour violation (EPHV; 17–19)
		Night violation (NV; 20–24)
		Other time (OT; 10–16)
	Spatial violation features	High-frequency location (H_LOC)
		Middle-frequency location (M_LOC)
		Low-frequency location (L_LOC)
**Violation Information**	Violation behavior features	Not riding on the non-vehicle lane (Type 1)
		Retrograding (Type 2)
		Red-light running (Type 3)
		Aggressive riding (Type 4)
		Speeding (Type 5)
		Drunk driving (Type 6)
		Overloading (Type 7)
		Other violations (Type 8)
	Punishment-related features	Cumulative violation frequency (CVF)
		Cumulative violation type frequency (CVTF)
		Cumulative prior penalty (CPP)
		Average prior penalty (APP)
		Maximum prior penalty (MPP)

**Table 2:** Adjusted confusion table of the gradient boost decision tree (GBDT) model. HR—high risk; NHR—non-high risk.

	NHR Rider	HR Rider	Threshold	Evaluation Index
**NHR Rider**	17,829	129	0.959	Recall = 0.596
**HR Rider**	263	388		Precision = 0.750
**NHR Rider**	17,682	276	0.909	Recall = 0.70
**HR Rider**	196	455		Precision = 0.622
